# Supplementary material for: Circularization restores signal recognition particle RNA functionality in Thermoproteus
Source: eLife. 2015 Oct 24;4:e11623. doi: 10.7554/eLife.11623 (PMC4731332; doi:10.7554/eLife.11623)
Supplement: Supplementary file 1. — This text-file contains two extended tables. Supplementary file 1A contains a list of predicted signal peptides for T. tenax. Supplementary file 1B lists all oligonucleotides and RNA sequences that were used in this study for cloning and RNA substrate generation. DOI: http://dx.doi.org/10.7554/eLife.11623.011 [file elife-11623-supp1.docx]

**Supplementary File**

Circularization restores signal recognition particle RNA functionality in *Thermoproteus*

André Plagens, Michael Daume, Julia Wiegel and Lennart Randau

**Supplementary File 1A - Signal peptide analysis of the *T. tenax* genome**.

All proteins of the *T. tenax* genome (NC_016070.1) were analyzed for putative signal peptides. Signal peptides were predicted using the tools PRED-TAT, PRED-Signal and SignalP 4.1. Proteins are listed that showed a positive hit for all three prediction tools. Shown are the ORF, the putative function of the protein and the predicted signal peptide sequence.

| **ORF** | **function** | **signal peptide** |
| --- | --- | --- |
| gi\|352681251 | Hypothetical protein | MKTRIIPIIIIGILALAGLAAA |
| gi\|352681256 | Thermopsin | MKRALALAMLLAAFIAAQPLQA |
| gi\|352681262 | Thermopsin precursor-like protein | MRALLLLAALALLPLLAA |
| gi\|352681266 | Hyaluronate lyase | MKLSRRDFLKASSLAAALSALNWSTLVKA |
| gi\|352681313 | Peptide ABC transporter substrate-binding protein | MRTSTIIAVVVVAIIVIAALA |
| gi\|352681377 | Thermopsin precursor | MRFPILLLLLVAIAYA |
| gi\|352681388 | Peptide ABC transporter substrate-binding protein | MSARKTTITAALIAVLVLSVYLAHA |
| gi\|352681420 | NurA-like nuclease | MNAKKIALILILVLVGSTLGAVVA |
| gi\|352681431 | Cobalamin biosynthesis protein | MRRLFIFLAIMALFSPIFG |
| gi\|352681439 | Ferredoxin:quinone oxidoreductase | MTLALLLIALGLIVIMA |
| gi\|352681448 | NADH-quinone oxidoreductase | MAWLIFLLLLIAGVAAMLIVPALFAPA |
| gi\|352681484 | ABC transporter substrate-binding protein | MILIGVIALVLVAIVFFLLQS |
| gi\|352681490 | Fe3+-hydroxamate ABC transporter substrate-binding protein | MRKTIYLGIAALVILALSAYYLS |
| gi\|352681527 | Hypothetical protein | MRAFALALLILAAVPALA |
| gi\|352681553 | Cytochrome b558/566 subunit A | MRHNPGSKLGRHKALLLLALVGIVGA |
| gi\|352681562 | Acid phosphatase | MRAPLALLLLAAFALAAA |
| gi\|352681564 | Hypothetical protein | MNKALVAAAVILAAAAVAAILMRTAPA |
| gi\|352681589 | Thermopsin precursor | MKAKIIAPILAILVLMALFMHQSA |
| gi\|352681596 | Hypothetical protein | MAGANRREALRLLGAAGLGALAGALAADLLAKPAPA |
| gi\|352681614 | Amino acid transporter | MGSLKLRGLFALSLAGILPAGA |
| gi\|352681620 | ABC transporter substrate-binding protein | MVSRRDALKIGASLVMGLGVGFLAGSLMPKGQQQPATA |
| gi\|352681707 | Branched-chain amino acid ABC transporter permease, LivM-like | MRPHIPVAIYASMIALALA |
| gi\|352681720 | Hypothetical protein | MRALALAAFFALAITAVFA |
| gi\|352681721 | ArsR family transcriptional regulator | MFNITVAVMLLPFTVYPINLA |
| gi\|352681724 | Hypothetical protein | MRAKLKAFFITFFPLLVFSIYIAA |
| gi\|352681771 | Sugar ABC transporter substrate-binding protein | MRASKLGLAVGIVVVLILIGAAAYFMSKTPPPTSSA |
| gi\|352681782 | NADH-ubiquinone oxidoreductase | MTGLALYISILLPMLLALTAYAAA |
| gi\|352681825 | Thermopsin-like protease | MRTLLLAMLLTAALALA |
| gi\|352681837 | Phosphatidylethanolamine-binding protein | MRRLLLIIAAVAVLAALLLA |
| gi\|352681847 | S-layer protein | MNKAWSFAIVALLLLLAQTEA |
| gi\|352681876 | Hypothetical protein | MPQTSKVIALILILVSVAVSINVYA |
| gi\|352681879 | Hypothetical protein | MRQYLGLLLTALSILILLPYTQA |
| gi\|352681909 | Cardiolipin synthase | MRYLFVLLVLSMFVFS |
| gi\|352681972 | Peptide ABC transporter permease | MSRALPVVLALWLAVYIFLASQS |
| gi\|352681976 | Hypothetical protein | MRLWWIGAALIAASLLPSVHFVAYA |
| gi\|352681983 | A1A0 ATP synthase subunit K | MKLRYITYLLLLSAVALA |
| gi\|352682001 | Conserved hypothetical protein | MLLIALALLALPSLLTSRSS |
| gi\|352682097 | Hypothetical protein | MRWMIELLLAVMLVALAVYA |
| gi\|352682161 | Uncharacterized conserved protein | MNKNGIIVMISILLITVGVLVLA |
| gi\|352682170 | Type II/IV secretion system membrane protein | MGVRGLISLRWLIVSA |
| gi\|352682173 | Secretion protein | MRGQGELVTIAAMLAVGAMAILIIQSVAHA |
| gi\|352682175 | Type II/IV secretion system protein | MDFELLTAATAVVVGALLAAAFLIRPLPPPA |
| gi\|352682177 | Type II/IV secretion system protein | MRRNPLIVATLFTAALIAAVLA |
| gi\|352682236 | Hypothetical protein | MKFAAASVLFSLIAFALALVMS |
| gi\|352682288 | Cobalt-precorrin-6A synthase | MLTLKRFGITTGAAAAAAAKA |
| gi\|352682296 | Hypothetical protein | MSLGLALLFAAAAVALVVFSLFATPPYLVVA |
| gi\|352682315 | Sugar-binding protein | MSPKTISIIVVIIIVAIAAAIFLTQQRGTPQAGA |
| gi\|352682325 | Type II/IV secretion system protein | MKFILLVLVGAALVFAQVYVFS |
| gi\|352682334 | Branched-chain amino acid ABC-transporter substrate-binding protein | MASKTGIIIGVVVLVIIIAAVAA |
| gi\|352682337 | Branched-chain amino acid ABC-transporter substrate-binding protein | MASKTGIIIGVVVLVIIIAAVAA |
| gi\|352682369 | Hypothetical protein | MIVFLMVFILFLLPVSVRFS |
| gi\|352682397 | Formate dehydrogenase | MTVSTTRRGFLKISALAALALGLPSSAQS |
| gi\|352682450 | Cell surface containing PKD-like repeats | MWAVAALALVLAAAGQFA |
| gi\|352682462 | Hypothetical protein | MYKLWAALILSAWAAWGFAIA |
| gi\|352682481 | Thermopsin | MRPALLPLLLLAASALA |
| gi\|352682505 | Conserved hypothetical protein | MQTPKYGLAKLALLLLLPAGWEA |
| gi\|352682556 | Hypothetical protein | MGRAYLLALVLMLIVTPAFA |
| gi\|352682573 | Uncharacterized membrane protein | MPIKVFGLLLALGALQFIAVVQIASA |
| gi\|352682578 | Major facilitator superfamily transporter | MRRGGRLGLVSLIVSLTIAA |
| gi\|352682608 | Cytochrome C-type biogenesis protein | MSIKPMRLLLAVLLMAALALA |
| gi\|352682617 | Hypothetical protein | MRLFVALIAVVALVSA |
| gi\|352682626 | Hypothetical protein | MIALLLAVVLGAYLASA |
| gi\|352682755 | Hypothetical protein | MRTVIALAVLLAVFLAYATS |
| gi\|352682954 | Maltose transport system substrate-binding protein | MRGLGKTTAIAIVVVIVLIIAVAAYF |
| gi\|352683007 | Putative NaMN:DMB phosphoribosyltransferase | MSTTFVIVVGTTDISLIPGITVAGA |
| gi\|352683058 | Conserved hypothetical protein | MHNPKTRVLLLMAIGLILIATMAHA |
| gi\|352683059 | Conserved hypothetical protein | MGVIALALYAIAPAVVT |
| gi\|352683110 | Peptidase S16 | MSKLYLVLVVILASLAPALVLSSLKPGTHMVVVGTTTINALA |
| gi\|352683115 | Peptidase S53 | MCHMDKLIAISILILISAVAVSA |
| gi\|352683198 | Conserved hypothetical protein | MRLKFLAMAIIGLTALA |
| gi\|352683240 | Conserved hypothetical protein | MALKQAVVLALVVTALFLSAQIQVSG |
| gi\|352683280 | Hypothetical protein | MYWAVTAALIIAMAMVIIVTIYTTGAMMQQA |
| gi\|352683282 | Uncharacterized protein with SCP/PR1 domains | MRRLALALTALLAVLLLLYA |

**Supplementary file 1B - Oligonucleotides and RNA sequences for cloning and RNA substrate generation.**

| **substrate** | **name** | **sequence** |
| --- | --- | --- |
| S-domain WT | S-d P1 for | GATCCTAATACGACTCACTATAGCCTCGGCCCTCGGGGCCTGCCCAGCCGGCGTACCCAAGCCGT  AGGGCTTGGCGGCGCCGGCGAAGGCGGCGAACC |
|  | S-d P1 rev | CCGGCGCCGCCAAGCCCTACGGCTTGGGTACGCCGGCTGGGCAGGCCCCGAGGGCCGAGGCTATA  GTGAGTCGTATTAG |
|  | S-d P2 for | AGGGGAGGTCCGGGAGGGAGCACCCTTAAGCCGCCGGGTAGTGCGTTCGAGGGGGCTCCGGGGCA |
|  | S-d P2 rev | AGCTTGCCCCGGAGCCCCCTCGAACGCACTACCCGGCGGCTTAAGGGTGCTCCCTCCCGGACCTC  CCCTGGTTCGCCGCCTTCG |
| S-domain Open | open P1 for | AGCTTTAATACGACTCACTATAGGAGCACCCTTAAGCCGCCGGGTAGTGCGTTCGAGGGGGCTCC  GGGGCGCCTCGGCCCTC |
|  | open P1 rev | CCGGAGCCCCCTCGAACGCACTACCCGGCGGCTTAAGGGTGCTCCTATAGTGAGTCGTATTAA |
|  | open P2 for | GGGGCCTGCCCAGCCGGCGTACCCAAGCCGTAGGGCTTGGCGGCGCCGGCGAAGGCGGCGAACCA  GGGGAGGTCCGGGAG |
|  | open P2 rev | GATCCTCCCGGACCTCCCCTGGTTCGCCGCCTTCGCCGGCGCCGCCAAGCCCTACGGCTTGGGTA  CGCCGGCTGGGCAGGCCCCGAGGGCCGAGGCGCC |
| S-domain GNAR | GNARm P1 for | GATCCTAATACGACTCACTATAGCCTCGGCCCTCGGGGCCTGCCCAGCCGGCGTACCCAAGCCGT  TGGGCTTGGCGGCGCCGGCGAAGGCGGCGAACC |
|  | GNARm P1 rev | CCGGCGCCGCCAAGCCCAACGGCTTGGGTACGCCGGCTGGGCAGGCCCCGAGGGCCGAGGCTATA  GTGAGTCGTATTAG |
|  | S-d P2 for + rev | see above |
| S-domain h8b | S-d P1 for + rev | see above |
|  | h8bm P2 for | AGGGGAGGTCCGGGAGGCTCCACCCTTAAGCCGCCGGGTAGTGCGTTCGAGGGGGCTCCGGGGCA |
|  | h8bm P2 rev | AGCTTGCCCCGGAGCCCCCTCGAACGCACTACCCGGCGGCTTAAGGGTGGAGCCTCCCGGACCTC  CCCTGGTTCGCCGCCTTCG |
| BHB RNA | BHB | GAUCGUCCCCGAAGGAGCCGUAGGGCUCCGGGAGAGCUAUAUC |
| Northern Blot | probe P1 | AGGCCCCGAGGGCCGAGGCTTCATCGCCCGCCAGACCGCCCACGT |
|  | probe P2 | TCCCGGACCTCCCCTGGTTCGCCGCCTTCGCCGGCGCCGCCA |
| PCR SRP19 | TTX_2083 for | AGCCATATGAGGAGAAAGGAG |
|  | TTX_2083 rev | TCTCTCGAGCCTCCTCAG |
| PCR SRP54 | TTX_0615 for | ATACATATGAGACCTCTGGCCGAG |
|  | TTX_0615 rev | GCTAAGCTTCGGCTGAGGAATG |
| PCR splicing endonuclease | TTX_1594 for | TGAGGAAGACCATGGGGAGGGGAGTTC |
|  | TTX_1594 rev | CATCTAAGCTTCTAAAGTTTCGCCCAC |
|  | TTX_1893 for | TCCCTCATATGGACGTCTTGGAGGAG |
|  | TTX_1893 rev | TGAGCCCTAGGAGCGTTTCAAATA |
